# Interferon-α Improves Phosphoantigen-Induced Vγ9Vδ2 T-Cells Interferon-γ Production during Chronic HCV Infection

**DOI:** 10.1371/journal.pone.0037014

**Published:** 2012-05-22

**Authors:** Eleonora Cimini, Cécile Bonnafous, Veronica Bordoni, Eleonora Lalle, Helene Sicard, Alessandra Sacchi, Giulia Berno, Cristiana Gioia, Gianpiero D’Offizi, Ubaldo Visco Comandini, Chrysoula Vlassi, Maria Rosaria Capobianchi, Federico Martini, Chiara Agrati

**Affiliations:** 1 Cellular Immunology Laboratory, National Institute for Infectious Diseases “Lazzaro Spallanzani” I.R.C.C.S, Rome, Italy; 2 Innate Pharma, Marseille, France; 3 Virology Laboratory, National Institute for Infectious Diseases “Lazzaro Spallanzani” I.R.C.C.S, Rome, Italy; 4 Clinical Department, National Institute for Infectious Diseases “Lazzaro Spallanzani” I.R.C.C.S, Rome, Italy; KAIST, Graduate School of Medical Science & Engineering, Republic of Korea

## Abstract

In chronic HCV infection, treatment failure and defective host immune response highly demand improved therapy strategies. Vγ9Vδ2 T-cells may inhibit HCV replication *in vitro* through IFN-γ release after Phosphoantigen (PhAg) stimulation. The aim of our work was to analyze Vγ9Vδ2 T-cell functionality during chronic HCV infection, studying the role of IFN-α on their function capability. IFN-γ production by Vγ9Vδ2 T-cells was analyzed *in vitro* in 24 HCV-infected patients and 35 healthy donors (HD) after PhAg stimulation with or without IFN-α. The effect of *in vivo* PhAg/IFN-α administration on plasma IFN-γ levels was analyzed in *M. fascicularis* monkeys. A quantitative analysis of IFN-γ mRNA level and stability in Vγ9Vδ2 T-cells was also evaluated. During chronic HCV infection, Vγ9Vδ2 T-cells showed an effector/activated phenotype and were significantly impaired in IFN-γ production. Interestingly, IFN-α was able to improve their IFN-γ response to PhAg both *in vitro* in HD and HCV-infected patients, and *in vivo* in *Macaca fascicularis* primates. Finally, IFN-α increased IFN-γ-mRNA transcription and stability in PhAg-activated Vγ9Vδ2 T-cells. Altogether our results show a functional impairment of Vγ9Vδ2 T-cells during chronic HCV infection that can be partially restored by using IFN-α. A study aimed to evaluate the antiviral impact of PhAg/IFN-α combination may provide new insight in designing possible combined strategies to improve HCV infection treatment outcome.

## Introduction

Most Hepatitis C virus (HCV) infections evolve in persistent infection, which may progress to fibrosis, cirrhosis, liver failure or even hepatocellular carcinoma [1]. Current standard therapy is based on a combination of pegylated (PEG)-IFN-α and ribavirin (RBV) and treatment response may be influenced by several virus-related factors such as HCV genotype and baseline titer of HCV RNA [2], [3]. A sustained virological response (SVR) occurs in approximately 80% of patients infected with HCV genotypes 2 or 3, and in approximately 45% for genotypes 1 or 4 [4]. New antiviral strategies are currently in development for HCV infection and include drugs targeting key viral enzymes such as NS3-4A and the NS5B RNA-dependent RNA polymerase [5]. Although effective, the use of these new antivirals seems associated to the selection of drug-resistant HCV variants, resulting in viral breakthrough. Thus, a combination between antivirals and standard treatment with IFN-α and RBV is therefore necessary [3], [6].

HCV persistence is mainly due to the failure of the host’s immune system to effectively and definitively clear the infection and generate protective cellular immunity. Indeed, marked quantitative and qualitative defects of HCV-specific CD8 T-cells have been described in HCV patients, correlated with innate immune cell impairment such as dendritic cell (DC) [7] and NK cells [8]–[10]. In this context, immune modulation could represent a promising strategy aimed to restore protective immune response, inducing a long lasting immunity, necessary to obtain viral eradication.

Among innate immune cells, Vγ9Vδ2 T-cells represent a good target for immunotherapy in infectious diseases [11], [12] for their multifaceted response capability [13]. They may specifically be activated both *in vitro* and *in vivo* by using phosphoantigens (PhAgs) [14] and aminobisphosphonates [15] without any MHC restriction. They elicit a dual antimicrobial activity, by directly affecting microbial replication [13], [16] and by modulating other cell subsets such as DC activation and maturation [17], neutrophils recruitment and activation [18], and Th1 immune response polarization [19].

Vγ9Vδ2 T-cells are involved in host response to many chronic viral infections, including HCV [13]. As observed in other chronic infection such as HIV [20], a decrease of peripheral Vγ9Vδ2 T-cell subset was observed associated to HCV infection [10]. Activated Vγ9Vδ2 T lymphocytes were found able to inhibit subgenomic HCV replication, and this effect was mediated mainly by IFN-γ release [21]. A role of recombinant IFN-γ on subgenomic HCV replication was also described [22]. Moreover, several studies showed that the combination of recombinant IFN-γ and IFN-α resulted in a strongly enhanced antiviral activity in the HCV replicon model, opening the way to new combined treatment approaches. Thus, IFN-γ induced by Vγ9Vδ2 T-cell stimulation could enhance standard treatment effectiveness.

In this work, phenotype and function of Vγ9Vδ2 T-cells were analyzed during chronic HCV infection, evaluating possible strategies aimed to improve their effector response. This approach was validated *in vivo* in a non-human primate model.

## Methods

### Ethics statement

This study was approved by the Ethics Committee of the National Institute for Infectious Diseases “L.Spallanzani”, and all enrolled individuals provided written informed consent.

All experiments on monkeys were performed in accordance with the recommendations of the Weatherall report, and were previously approved by the regional ethical committee (Comité Régional d’Ethique en Matière d’Expérimentation Animale de Strasbourg: C.R.E.M.E.A.S.) (number approval: AL/01/01/01/06).

### Patients

24 HCV-infected patients (16 males and 8 females, mean age: 54.9±10.7) naïve to treatment, and 35 healthy age-matched individuals (HD, 25 males and 10 females, mean age: 50.3±13.2), were recruited at the INMI L. Spallanzani. Patients clinical features are described in Table 1. This study was approved by the Ethics Committee of the Institute, and all enrolled individuals provided written informed consent.

**Table 1:** Main clinical features of Healthy Donors (HDs) and HCV patients.

Group	Gender	Age	AST	ALT	HCV	HCV
	(M/F)	(years)	(mU/ml)	(mU/ml)	Genotype	V_L_ (log)
HD	25/10	50.3±13.2	22.8±8.2	21.6±7.1	n.t.	n.t.
(n = 35)						
HCV	16/8	54.9±10.7	60.7±34.4	55.0±39.6	1 (n = 11)	5.95±0.59
(n = 24)					2 (n = 5)	
					3 (n = 5)	
					4 (n = 3)	

n.t.: not tested.

### Plasma HCV quantification and genotyping

Plasma HCV-RNA levels were assayed by Abbott RealTimeHCV assay (Abbott Laboratories. Abbott Park, Illinois, U.S.A.). Moreover, HCV genotype was determined by Abbott RealTime HCV Genotype II Amplification Reagent kit.

### Lymphocytes isolation and γδ T cell purification

Peripheral blood mononuclear cells (PBMC) were isolated by Lympholyte (Cedarlane, Canada). In selected experiments, γδ T-cells were purified from PBMC by immunomagnetic separation using anti-γδ-conjugated magnetic microbeads (Miltenyi Biotec, Germany). The purity of cells fraction was >95% in all experiments, as measured by flow cytometry analysis (data not shown).

### Vγ9Vδ2 T-cell phenotyping

Phenotypic analysis of Vδ2 T-cells from HCV and from HD was performed by flow cytometry. Specifically, Vδ2 T-cell subsets were analyzed by using the following monoclonal antibodies: anti- Vδ2 FITC (clone IMMU389), anti-CD3 PerCP-PC5 (clone UCHT-1), from Beckman Coulter (Immunotech, France); anti-CD27 APC (clone L128), anti-CD45RA CY-Chrome (clone HI100), anti-CD69 APC-Cy7 (clone FN50), anti-CD25 APC (clone M-A251) from BD Biosciences (San Jose, CA, USA). Briefly, thawed PBMC (1×10^6^ cells/ml) were incubated with mAbs cocktail for 15 min a 4°C, washed twice with wash buffer (PBS 1×, 0.1% NaN_3,_ 1% BSA) and fixed with 1% paraformaldehyde (PFA, Sigma, St. Louis, MS). Samples acquisition and data analysis were performed by a FACS Canto II flow cytometer (Becton Dickinson) by using Diva software.

### Cytokines production

Cytokine production by Vγ9Vδ2 T-cells was tested by using a synthetic PhAg (IPH1101, Innate-Pharma, France) able to specifically activate only Vγ9Vδ2 T-cells [14]. Specifically, purified Vγ9Vδ2 T-cells from HD (n = 6) and PBMC from HD (n = 35) or HCV-patients (n = 24) were stimulated with single PhAg (IPH1101: 3 µM), single IFN-α-2b (100 IU/ml, Schering-Plough, Belgium) or PhAg plus IFN-α-2b combined stimulation; IFN-γ production was evaluated after 18 hours by ELISA test (Thermo Scientific, USA).

Moreover, in selected HCV and HD, the frequency of IFN-γ-producing Vγ9Vδ2 T-cells was monitored. Briefly, PBMC were stimulated for 18 h with PhAg or PhAg/IFN-α in the presence of Brefeldin A (10 µg/ml) (Serva, Germany) to block cytokine secretion. Intracellular staining was performed by staining cells for 15 minutes at 4°C with anti-Vδ2 FITC antibody; after washing, cells were fixed with 1% PFA (Sigma, St. Louis, MS) and stained at room temperature with an APC-labeled IFN-γ specific antibody (clone B27), in permeabilizing solution (PBS 1×, 0.1% NaN_3,_ 1% BSA, 0.5% saponin). After washing (PBS 1×, 0.1% NaN_3,_ 1% BSA, 0.1% saponin), cells were acquired by flow cytometer (FACS Canto II flow cytometer) and data were analyzed by using Diva software. The frequency of IFN-γ-producing Vγ9Vδ2 T-cells and the IFN-γ MFI (Median Fluorescence Intensity) were compared between HD and HCV-infected patients.

### In vivo drug administration and cytokine quantification in animal system

8 naïve cynomologus macaques (*Macaca fascicularis*) were included in the study: 6 animals were purchased from Noveprim (Ferney S.E, Mahebourg, Mauritius) and 2 from CDP (ULP Strasbourg, France). Animal welfare conditions conformed to the European requirements, comprising monitored temperature, humidity, air change, and lighting cycle. All experiments were previously approved by the regional ethical committee (Comité Régional d’Ethique en Matière d’Expérimentation Animale de Strasbourg: C.R.E.M.E.A.S.) (number approval: AL/01/01/01/06). At the beginning of the study, body weights ranged from 2.2 to 5.8 kg. In order to avoid suffering, animals were anaesthetized with Ketamine 1000 ND (10 mg/kg IM) before any procedure. Group 1 (4 animals) was injected s.c. with 3 mg/Kg of IPH1201 (C-HDMAPP) a second generation synthetic PhAg able to specifically activate Vγ9Vδ2 T-cells (Innate-Pharma, France) (solution 4%, borate buffer). Group 2 (4 animals) was injected subcutaneously (s.c.) with 3 mg/Kg of IPH1201 and with 27 µg/animal s.c. of Interferon α-2a pegylated, Pegasys® (Roche). The dose of Pegasys is the same as that used in the clinical care of HCV patients. Blood samples were collected before and after 4, 8, 12, 16, 20, 24, 28 hours after treatment, and sera were stored for further analysis. IFN-γ plasma levels were analyzed by ELISA (Biosource).

### Analysis of IFN-γ-mRNA level and stability

RNA from purified γδ T cells was extracted with Trizol reagent (Invitrogen, USA). One µg total RNA was reverse transcribed by TaqMan Reverse Transcription Reagent kit (Applied Biosystems, USA) according to manufacturer’s instructions. IFN-γ-mRNA level was quantified by qPCR performed using Taqman 2× PCR Master mix (Applied Biosystems, USA) and a 7900 HT Fast Real-Time PCR system machine by using primers and probe sets for IFN-γ-mRNA and β-actin as described in [23]. Results are expressed as normalized to β-actin expression.

mRNA stability was evaluated by adding 10 µg/ml actinomycin D (ActD) after 18 hours of PhAg (IPH1101: 3 µM) or IFN-α (100 IU/ml) stimulation. IFN-γ-mRNA levels were evaluated by qRT-PCR just before Actinomycin addiction (t0), and after 30 and 120 minutes of culture, and expressed as normalized to β-actin. mRNA stability was evaluated by calculating half-life times of IFN-γ-mRNA by linear regression (GraphPad Prism).

### Statistical analysis

Statistical significance was determined by GraphPad Prism software (GraphPad). Differences between groups were evaluated by non parametric Mann-Whitney test; Wilcoxon test was used when comparing different culture conditions of the same cells. Tests were considered significant when p<0.05. IFN-γ-mRNA half life was evaluated by linear regression.

## Results

### Vγ9Vδ2 T-cell phenotype and function in HCV-infected patients

**Figure 1:**
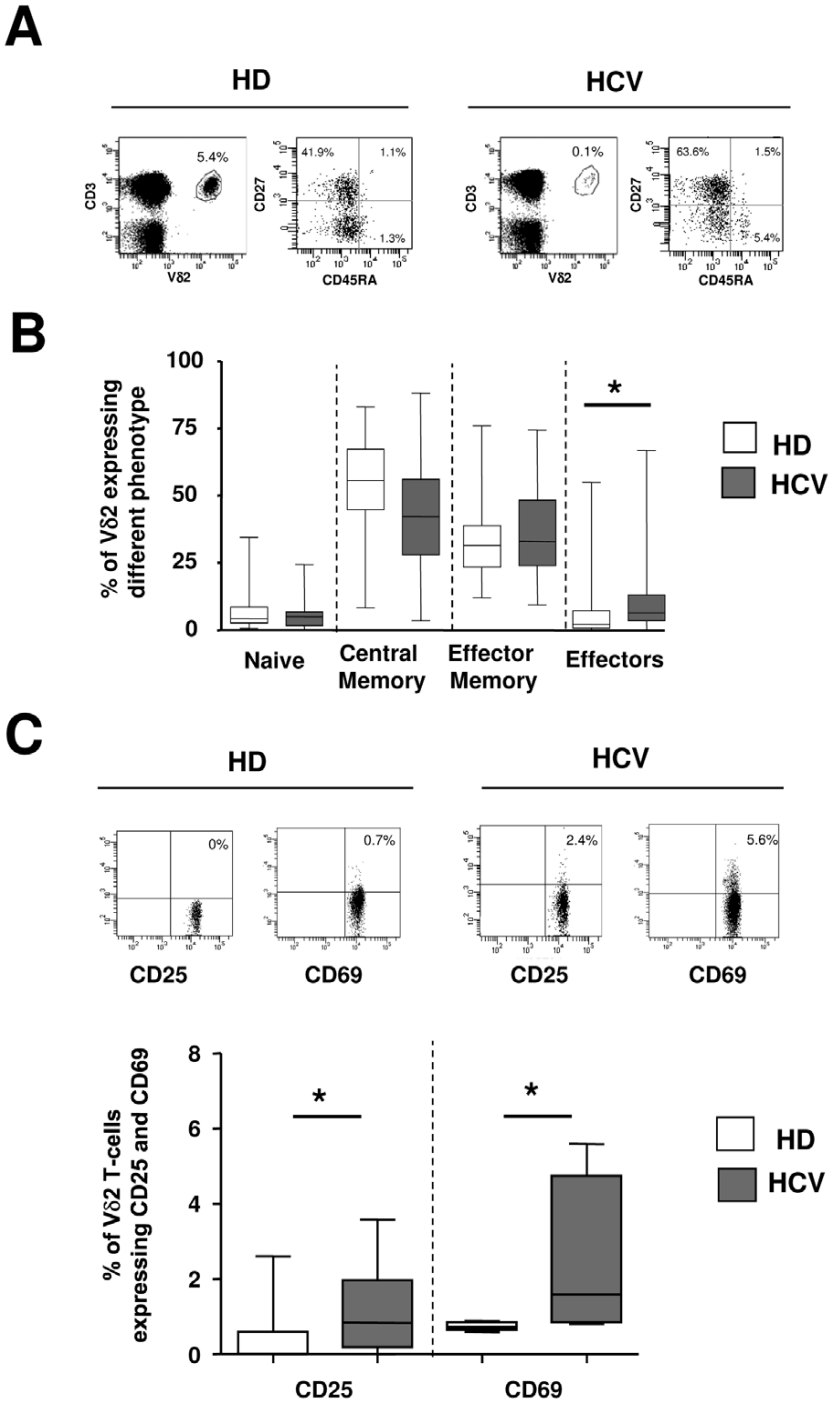
Chronic HCV infection induces an increase in activated/effectors Vγ9Vδ2 T-cells. (A) Representative flow cytometry panels on Vγ9Vδ2 T-cells frequency and differentiation profile are shown for one healthy donor and one HCV-infected patient. Differentiation was analyzed by monitoring CD27 and CD45RA expression. Naïve: CD45RA+CD27+; Central Memory: CD45RA-CD27+; Effector Memory: CD45RA-CD27-; Effectors: CD45RA+CD27-. (**B**) Statistical analysis of Vγ9Vδ2 T-cell differentiation profile from HD (white boxes, n = 35) and HCV (grey boxes n = 24) was performed by Mann-Whitney test. *p<0.05. (**C**) Representative flow cytometry panels on CD25 and CD69 expression on Vγ9Vδ2 T-cells are shown for one healthy donor and one HCV-infected patient. (**D**) Statistical analysis of CD25 and CD69 expression on Vγ9Vδ2 T-cells from HD (white boxes, n = 35) and HCV (grey boxes n = 24) was performed by Mann-Whitney test. *p<0.05.

Vγ9Vδ2 T-cell subsets were analyzed in 24 HCV-infected patients (HCV), naïve to treatment, and compared with 35 healthy donors (HD). In chronic HCV patients a slight but significant decrease in circulating Vγ9Vδ2 T-cell frequency was observed [HCV: median 1.140% (IQR: 0.49–2.16) vs. HD: 1.770% (1.080–2.290), p = 0.0362, Figure 1A], confirming previous results [10]. Moreover, Vγ9Vδ2 T-cell differentiation profile showed a significant increase in Vγ9Vδ2 effector cells (CD45RA+CD27-) in HCV patients [HCV: median 6.5 (IQR: 3.5–13.0) vs. HD: 2.2 (0.7–7.2), p = 0.0214], suggesting that chronic HCV infection induced Vγ9Vδ2 T-cell differentiation toward effector functions (Figure 1A–B). Moreover, CD25 and CD69 expression on Vγ9Vδ2 T-cells were slightly but significantly increased in HCV patients as compared to HD [CD25: HCV median 0.8% (IQR 0.2–2.0) vs. HD 0.0% (IQR: 0–0.6), p = 0.0435; CD69: HCV median 1.6 (IQR 0.9–4.8) vs. HD 0.7 (IQR: 0.6–0.9), p = 0.0317, Figure 1C–D], suggesting that HCV-induced chronic inflammation may increase basal activation of these cells.

**Figure 2:**
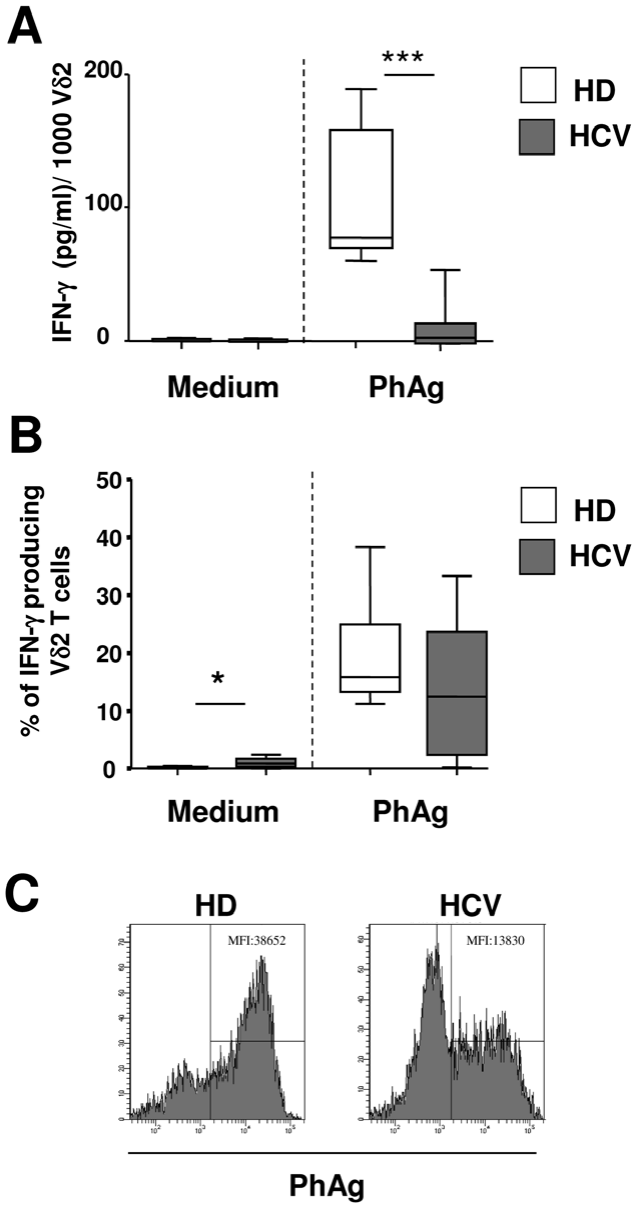
Chronic HCV infection induces a strong impairment in IFN-γ production. (A) A quantitative analysis of IFN-γ produced by unstimulated and PhAg-stimulated Vγ9Vδ2 T-cells from HD (n = 20, white boxes) and HCV (n = 24, grey boxes) was performed by ELISA assay. (**B**) The frequency of IFN-γ-producing Vγ9Vδ2 T-cells after PhAg stimulation was analyzed by intracellular staining and flow cytometry. Statistical analysis was performed by Mann-Whitney test, *p<0.05; ***p<0.0001. (**C**) Representative flow cytometry histograms of IFN-γ MFI (Median Fluorescence Intensity) produced by Vγ9Vδ2 T-cells after PhAg stimulation are shown for one healthy donor and one HCV-infected patient.

It is well known that PhAgs specifically activate only Vγ9Vδ2 T-cell subset inducing IFN-γ release [14]. Thus, IFN-γ production by Vγ9Vδ2 T-cells from HCV-infected patients (n = 24) and HD (n = 20) was analyzed by stimulating PBMC for 18 hours with a Vγ9Vδ2 T-cells specific PhAg (3 *μ*M). The amount of IFN-γ released in supernatants was measured by ELISA (Figure 2A). PBMC from HCV patients showed a profound impairment in IFN-γ production after PhAg stimulation [HCV: median 4.4 pg/ml (IQR: 0.0–14.9) vs. HD: 77.1 pg/ml (69.5–158.1), p<0.0001].

Finally, we wondered whether the lower IFN-γ production observed in chronic HCV infected patients was the result of a decreased frequency of IFN-γ-producing Vγ9Vδ2 T-cells, or of a reduced amount of IFN-γ produced by each cell. To this aim, we quantified the frequency of IFN-γ-producing Vγ9Vδ2 T-cells by intracellular staining and flow cytometry. As shown in Figure 2B, no statistically significant differences in the frequency of PhAg-stimulated Vγ9Vδ2 T-cells between HD and HCV was observed, suggesting that HCV infection reduced the amount of IFN-γ produced by each responding cells. Indeed, the IFN-γ MFI after PhAg stimulation was lower in HCV patients than in healthy donors [HCV-MFI: 13830 (IQR: 12240–18440) vs. HD-MFI: 40340 (IQR: 38652–51900), p<0.05, Figure 2C], confirming a reduced capability of each responding cells to produce IFN-γ. Moreover, a slight percentage of Vγ9Vδ2 T-cells from HCV-infected patients were able to produce IFN-γ in the absence of antigenic stimulation [HCV: median 0.9% (IQR: 0.35–1.75) vs. HD: 0% (0–0.34), p<0.05], Figure 2b), confirming an activated/effector phenotype.

### IFN-α improves in vitro and in vivo Vγ9Vδ2 T-cell responsiveness to PhAg stimulation in HD and in non-human primates

**Figure 3:**
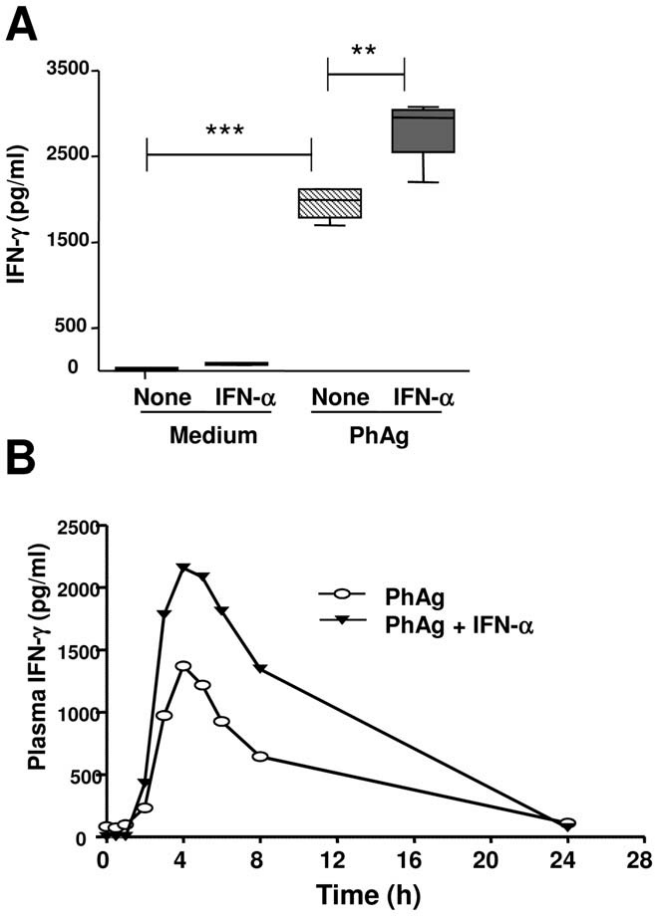
IFN-α improves PhAg-induced IFN-γ production by Vγ9Vδ2 T-cells in HD and in non-human primates. (**A**) A quantitative analysis of IFN-γ production was performed *in vitro* by stimulating purified Vγ9Vδ2 T-cells from 6 HD by ELISA after medium (white boxes), IFN-α (grey boxes), PhAg (hatched boxes) and PhAg/IFN-α (dark grey boxes) stimulation. Statistical analysis was performed by Mann-Whitney test, **p<0.01 ***p<0.0001. (**B**) Plasma IFN-γ levels from *in vivo* PhAg (white dots, n = 4) and PhAg/IFN-α (black dots, n = 4) treated monkeys was quantified by ELISA test.

In order to evaluate whether IFN-α could improve Vγ9Vδ2 T-cell responsiveness to PhAg, purified Vγ9Vδ2 T-cells were stimulated with single PhAg (3 µM), single IFN-α (100 IU/ml) and combined (PhAg/IFNα) for 18 hours. At the end of incubation IFN-γ released by Vγ9Vδ2 T-cells was evaluated by ELISA test (Figure 3A). As shown in Figure 3A, IFN-α was able to improve IFN-γ release by Vγ9Vδ2 T-cells [PhAg: median 1996 pg/ml (IQR: 1791–2115) vs. PhAg/IFNα: 2953 (2550–3042), p<0.05]. Moreover, dose response experiments showed that IFN-α did not modify EC_50_ of PhAg but it was able to induce a dose dependent increase of IFN-γ production (data not shown).

To verify if IFN-α could also improve *in vivo* Vγ9Vδ2 T-cell responsiveness to PhAg stimulation**,** non-human primates (*M. Fascicularis*) were injected with 3 mg/Kg s.c. of PhAg (Group 1, n = 4) or with 3 mg/Kg of PhAg s.c. and 27 µg/animal s.c. of PEG-IFN-α (Group 2, n = 4). Plasma IFN-γ levels were analyzed before administration, and after 4, 8, 12, 16, 20, 24, 28 hours. As shown in Figure 3B, no IFN-γ was found before treatment, and a single injection of PhAg resulted in an increase in plasma IFN-γ level, reaching a peak after 4 hours, declining afterwards. Interestingly, the combined injection of IFN-α and PhAg was able to strongly increase IFN-γ release (C_max_ PhAg: 1,370 pg/ml vs. C_max_ PhAg/IFN-α: 2,155 pg/ml), showing that PhAg/IFN-α combination is able to boost *in vivo* IFN-γ production.

### IFN-α improves in vitro Vγ9Vδ2 T-cell responsiveness to PhAg stimulation in HCV-infected patients

**Figure 4:**
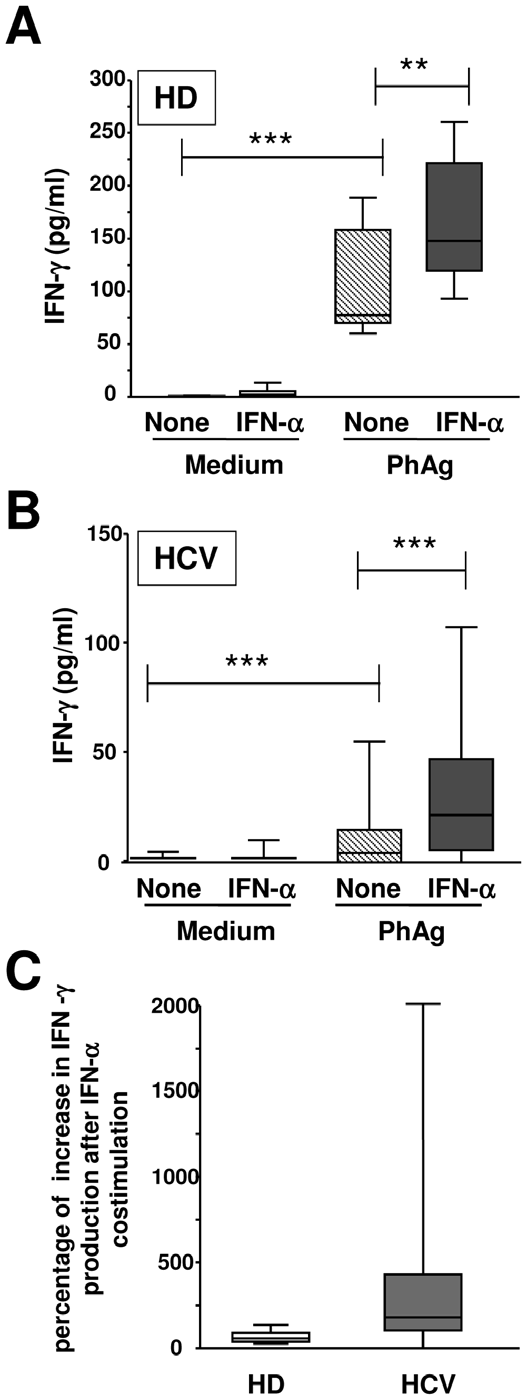
IFN-α improves *in vitro* PhAg-induced IFN-γ production of Vγ9Vδ2 T-cells in HCV patients. (**A,B**) A quantitative analysis of IFN-γ production was performed in HD (n = 35, Panel A) and in HCV (n = 24, Panel B) by ELISA after medium (white boxes), IFN-α (grey boxes), PhAg (hatched boxes) and PhAg/IFN-α (dark grey boxes) stimulation. Statistical analysis was performed by Mann-Whitney test, **p<0.01 ***p<0.0001. (**C**) The percentage of increase in IFN-γ production after combined PhAg/IFN-α respect to single PhAg stimulation was compared between HD (white bar) and HCV patients (grey bar). Statistical analysis was performed by Mann-Whitney test, **p<0.01.

Since IFN-α was able to improve Vγ9Vδ2 T-cell responsiveness in HD, we wondered whether it can restore the impaired functional activities of Vγ9Vδ2 T-cells during chronic HCV infection. To this aim, IFN-γ production (Figure 4) after 24 hours of single PhAg (3 µM), single IFN-α (100 IU/ml), or combined (PhAg/IFNα) *in vitro* stimulations was evaluated on PBMC from 24 HCV-infected patients and 35 HD. Figure 4 shows that IFN-α was able to increase IFN-γ production by Vγ9Vδ2 T-cells after PhAg stimulation both in HD [HD: PhAg: median 77.1 (IQR: 69.5–158.1) vs. PhAg/IFNα: 147.9 (119.9–221.6), p = 0.004, Figure 4A] and in HCV-infected patients [HCV: PhAg: median 4.4 (IQR: 0.0–14.9) vs. PhAg/IFNα: 21.8 (5.6–47.4), p<0.0001, Figure 4B]. Notably, in HCV-infected patients, IFN-γ production by Vγ9Vδ2 T-cells after PhAg/IFN-α stimulation did not reach the level found in HD. Nevertheless, the relative impact of IFN-α in improving individual Vγ9Vδ2 T-cell responsiveness was higher in HCV-infected patients than in HD (Figure 4C).

### IFN-α improves and stabilizes PhAg-induced IFN-γ –mRNA

**Figure 5:**
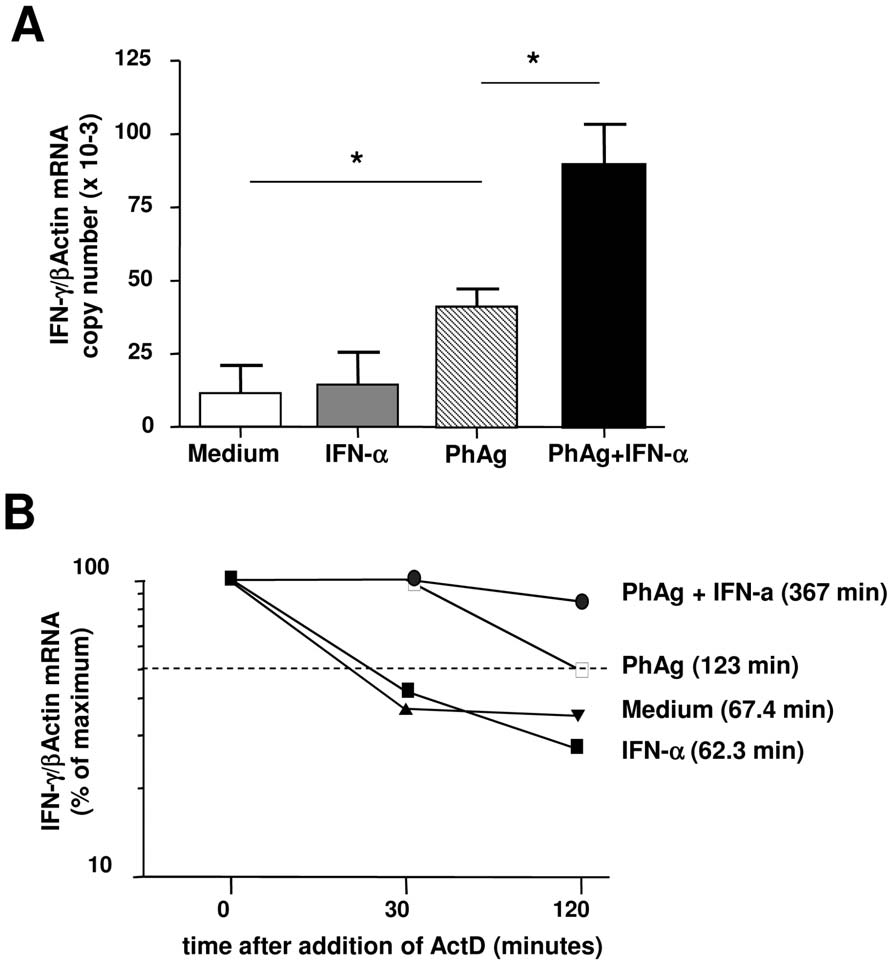
IFN-α and PhAg synergistically induce and stabilize IFN-γ-mRNA. (**A**) IFN-γ-mRNA levels in purified Vγ9Vδ2 T-cells were evaluated by TaqMan qRT-PCR after 18 hours of medium (white bar), IFN-α (grey bar), PhAg (hatched bar), and PhAg/IFN-α (black bar) stimulation (n = 4). Statistical analysis was performed by Mann-Whitney test. *p<0.05. (**B**) IFN-γ-mRNA stability over time was measured by adding actinomycin-D to cultures after 18 hours of stimulation with medium (black triangles), IFN-α (black squares), PhAg (white squares), PhAg/IFN-α (black circles). The number of copies of IFN-γ-mRNA was normalized in respect to β-actin. Results from one representative donor are shown. Times in brackets represent mRNA half life evaluated by linear regression.

A quantitative analysis of IFN-γ-mRNA after PhAg and PhAg/IFN-α stimulations of purified Vγ9Vδ2 T-cells was performed by qRT-PCR (Figure 5A). As expected, PhAg induced a significant increase in IFN-γ-mRNA [IPH1101: 40.8 (IQR: 33.7–48.9) vs. medium: 2.4 (1.5–21.5), p = 0.0286], while IFN-α alone did not induce any IFN-γ-mRNA. Interestingly, the combined stimulation by PhAg and IFN-α strongly enhanced IFN-γ-mRNA expression (PhAg/IFN-α: 84.4 (68.5–110.1) vs PhAg: 40.8 (IQR: 33.7–48.9), p = 0.0286), suggesting that IFN-α increased PhAg-induced IFN-γ- transcription (Figure 5A).

IFN-γ-mRNA persistence was studied by blocking transcription with actinomycin D after 18 hours of stimulations (Figure 5B). We defined 100% IFN-γ-mRNA as the amount, normalized to β-actin mRNA, found after 18 hours of stimulation, just before actinomycin D addition. IFN-γ-mRNA level was measured after 30 and 120 minutes after actinomycin D addition, and mRNA half-life was calculated by regression analysis. As reported in Figure 5B, IFN-γ-mRNA from non stimulated and IFN-α stimulated Vγ9Vδ2 T-cells rapidly decreased after the addiction of actinomycin D (half-life: 67.4 and 62.3 min. respectively). Differently, IFN-γ-mRNA induced by PhAg stimulation persisted much longer (half-life: 123 min.), while the combined stimulation by PhAg and IFN-α highly improved IFN-γ-mRNA half life (367 min.), suggesting that the combined action of PhAg and IFN-α could be at least partially mediated by an increased stabilization of IFN-γ mRNA.

## Discussion

Main aim of our work was to study the effects of chronic HCV infection on Vγ9Vδ2 T-cell phenotype and function, and on possible strategies aimed to improve their effector activity.

Chronic HCV infection induced a slight but significant decrease in the frequency of Vγ9Vδ2 T-cells. An increased liver tissue compartmentalization of these cells may represent an additional factor [24]. Differentiation and activation profile analysis of Vγ9Vδ2 T-cells showed an increase in circulating effector and activated cells. These data may be explained in the context of a chronic infection leading to a persistent stimulation of immune cells, driving their activation and differentiation. In our patients, no correlation was found between Vγ9Vδ2 T-cells dysfunction and any clinical parameter.

Vγ9Vδ2 T-cells play a pivotal role in viral infections, for their ability to mediate broad antiviral and immunomodulating activities [13], [19]. Specifically, antiviral role of activated Vγ9Vδ2 T-cells, mainly mediated by IFN-γ release, has been demonstrated for several viruses such as coronavirus [25], orthopoxvirus [26], HIV [27], and HCV [21]. In our work, a severe functional inability of Vγ9Vδ2 T-cells to produce IFN-γ was shown in HCV patients, independently from viral load and genotype. Other innate immune cells are known to show quantitative and qualitative defects during chronic HCV infection such as DC [28] and NK cells [8]–[10], that could be associated to adaptive immune response dysfunction and/or exhaustion [28]. In this context, a complex network of different signals can act to induce immune cell exhaustion, such as chronic inflammation, persistent antigen stimulation, and/or direct viral effects [29]. Chronic inflammation and persistent antigen stimulation, as observed during HIV infection, may result in Vγ9Vδ2 T-cell exhaustion and anergy through activation-induced cell death [30], or through a decrease in Vγ9Vδ2 T-cells response by down-modulating CD3-ξ chain expression [31]. Finally, although controversial [32], [33], a possible direct HCV-driven inhibition of NK cell function through HCV-E2/CD81 binding has been reported [34]. Interestingly, CD81 expression by γδ T-cells was previously reported [35]. A study aimed to define cellular and molecular mechanisms involved in Vγ9Vδ2 T-cells exhaustion during chronic HCV infection may be useful to evaluate possible strategies to restore their activity.

The main result of our work is the demonstration that Vγ9Vδ2 T-cell function may be improved by IFN-α both in HD and in HCV-infected patients, resulting in a higher IFN-γ production. A first demonstration that type-I IFN may be sensed by Vγ9Vδ2 T-cells was reported by Kunzmann *et al.*, showing an increase of CD69 after IFN-α treatment [36]. We confirmed this observation (data not shown) and showed the ability of IFN-α to increase Vγ9Vδ2 T-cell response to PhAgs stimulation in terms of IFN-γ production both in HD and in HCV-infected patients. In particular, the significant impairment of Vγ9Vδ2 T-cells in HCV-infected patients did not allow to obtain their complete restoration by IFN-α. Nevertheless, individual relative impact of PhAg/IFN-α co-stimulation was found much higher in HCV patients, due to the very low level of responsiveness to PhAgs. Thus, the possibility to restore IFN-γ production *in vivo* by combining standard IFN-α treatment and PhAg stimulation may have a positive impact on HCV inhibition. Indeed several reports show that IFN-α and IFN-γ may synergistically inhibit HCV replication *in vitro* [22], [37], [38] and this effect is also reported for other viruses [39]. Nevertheless, a study aimed to evaluate the antiviral impact of PhAg/IFN-α combination is ongoing and may validate new combined treatment strategies. Interestingly, PhAg-activated Vγ9Vδ2 T-cells are able not only to produce IFN-γ but also to deploy many different response pathways, such as DC activation [17], and neutrophils recruitment/activation [18], thus improving the overall protective immune response capability. Noteworthy, IFN-α effect on PhAg/response was found also *in vivo* in pre-clinical trials on non-human primates, inducing an increase in IFN-γ amount in animals sera. A time-course study of *in vivo* IFN-α treatment on Vγ9Vδ2 T-cell responsiveness to PhAg in HCV-infected patients is currently in progress.

About possible mechanisms mediating this improvement, we found that IFN-α acts by increasing IFN-γ-mRNA persistence, that may result in increased IFN-γ translation levels. Similar observations were reported on NK cells, as IFN-γ production after IL-12 and IL-18 stimulation was regulated by mechanisms involving IFN-γ-mRNA stabilization [40]. Indeed, mRNA stabilization is now considered as one of the main post-transcriptional control mechanisms responsible for the initiation and resolution of inflammation [41].

In recent years, a new attention on new direct antiviral drugs for chronic HCV infection is growing. Nevertheless, a combination of these new treatments with IFN-α/Ribavirin seem necessary to avoid the emergence of drug resistance [6], [42]. The definition of other combined immunomodulating approaches may contribute to optimize the antiviral response. In this context Vγ9Vδ2 T-cells may represent a good target of immunomodulating strategies for their ability to be easily activated *in vivo* by PhAgs [12], [43]–[45] without HLA restriction [46] and to orchestrate a complex network of antiviral and immunomodulating activities [17]–[19]. We show here for the first time that IFN-α, currently used in standard therapy, is able to improve Vγ9Vδ2 T-cell responsiveness in HCV patients. This, and the finding that IFN-γ can act synergistically with IFN-α to inhibit HCV replication [22], [37], [38], strengthen the rational for testing combined standard antiviral and immunostimulating therapeutical strategies. To this aim, future *in vivo* studies on HCV-infected non-human primates aimed to define the antiviral capability of the combined treatment are necessary both to assess safety and antiviral effectiveness of this combined approach, and to disclose the cellular/molecular mechanisms involved.
